# Zinc finger Asp-His-His-Cys palmitoyl -acyltransferase 19 accelerates tumor progression through wnt/β-catenin pathway and is upregulated by miR-940 in osteosarcoma

**DOI:** 10.1080/21655979.2022.2040827

**Published:** 2022-03-17

**Authors:** Shuhong Liang, Xiaojian Zhang, Jun Li

**Affiliations:** Department of Pharmacy, The First Affiliated Hospital of Zhengzhou University, Zhengzhou, P.R. China

**Keywords:** ZDHHC19, miR-940, osteosarcoma, wnt/β-catenin pathway

## Abstract

Osteosarcoma (OS) is the most frequent malignant primary bone tumor in children and young adults. Zinc finger Asp-His-His-Cys palmitoyl-acyltransferase 19 (ZDHHC19) is a key enzyme in protein palmitoylation and plays crucial roles in tumor progression. However, its expression profile and biological function in OS have been unclear. In the present study, the expression level of ZDHHC19 in OS cell lines was determined by qRT-PCR and Western blot. The effect of ZDHHC19 in cell growth, invasion and migration was analyzed by CCK8, EDU, transwell, wound healing assay *in vitro*, and xenograft tumor model *in vivo*. In addition, bioinformatics analysis was used to explore the potential mechanism of ZDHHC19 in OS. Furthermore, the luciferase reporter assay was conducted to determine the direct binding between miR-940 and ZDHHC19. We discovered that ZDHHC19 was overexpressed in OS cells compared with the normal cells. The functional investigation demonstrated that ZDHHC19 silencing could inhibit proliferation, invasion and migration of OS *in vitro* and suppress tumorigenicity and lung metastasis in a xenograft model *in vivo*. Mechanistically, we identified that ZDHHC19 was a direct target of miR-940 and forced ZDHHC19 expressions partially rescue the suppression of proliferation, migration and invasion induced by miR-940. Moreover, bioinformatics analysis combined with validation experiments revealed that activating wnt/β-catenin pathway contributed to the pro-oncogenic effect induced by ZDHHC19. Furthermore, rescue experiments further verified that miR-940/ZDHHC19 axis regulated wnt/β-catenin pathway. Overall, these findings indicated that miR-940/ZDHHC19 axis played a significant role in OS progression and might be considered as a novel target for OS treatment.

**Abbreviations**: OS, osteosarcoma; miRNAs, microRNAs; 3’-UTR, 3’- untranslated region; TARGET, Therapeutically Applicable Research To Generate Effective Treatments; qRT-PCR, quantitative real-time PCR; IHC, Immunohistochemistry; GSVA, Gene Set Variation Analysis; GSEA, Gene Set Enrichment Analysis; KEGG, Kyoto Encyclopedia of Genes and Genomes;

## Introduction

Osteosarcoma (OS) is the most frequent malignant primary bone tumor occurring during young adults and children [1]. It originates from mesenchymal stem cells and composed almost 60% of all sarcoma cases [2,3]. The 5-year survival rate of OS is approximately 20% because of early metastasis, relapse and drug resistance [4]. Accordingly, further identification of the mechanisms of OS occurrence and exploration of treatment targets are pressing needs.

Palmitoylation is a remarkably significant and abundant event in post-translational modification [5]. The process of protein S-palmitoylation is catalyzed by a family of zinc finger Asp-His-Cys domains containing palmitoyltransferases, which includes 23 human DHHC proteins [6]. Increasing evidence has identified that the DHHC family serves a crucial role in many normal physiology processes as well as diseases, including cancers [7]. ZDHHC19, a key enzyme in the DHHC family, has been reported to be upregulated in human lung squamous cell carcinomas, and high ZDHHC19 expression could promote STAT3 activation through S-Palmitoylation [8]. However, the expression profile and biological function of OS have not been disclosed.

MicroRNAs (miRNAs) are a group of endogenous noncoding RNAs with 18–25 nucleotides long [9,10]. It could result in the degradation of the mRNA and the suppression of translation by binding to the 3’- untranslated region (3’-UTR) of target messenger RNA [10]. Emerging studies have emphasized that miRNAs are involved in multiple processes related to tumorigenesis and development, such as angiogenesis, proliferation, invasion and apoptosis [11–13]. MiR-940 was identified as a tumor inhibitor, and played an important role in various tumors, including prostate cancer [14], pancreatic ductal adenocarcinoma [15], hepatocellular carcinoma [16] and so on. In osteosarcoma, miR-940 was reported to suppress cell invasion and migration through SFRP1-mediated wnt/β-catenin pathway [17]. However, its molecular mechanism and specific function in osteosarcoma have not been determined completely.

In this study, we identified that ZDHHC19 was upregulated in OS cell lines, and ZDHHC19 overexpression was related to poor prognosis. Meanwhile, ZDHHC19 could accelerate proliferation, invasion and migration of OS cells through activating wnt/β-catenin pathway. Further, ZDHHC19 was a direct target of miR-940 and could reserve the inhibiting action of miR-940 in OS. These findings demonstrate the possibility of a distinct role for miR-940/ZDHHC19 axis in the OS treatment.

## Materials and methods

### Cell culture

The normal human bone cells (HFOB and HOBC) and three OS cell lines (U2OS, MG63 and 143B) were purchased from Procell cell bank (Wuhan, China). All cells were maintained in a high-glucose DMEM (Invitrogen, CA) which was supplemented with 10% fetal bovine serum (Invitrogen, CA) at 37°C in a humidified atmosphere containing 5% CO_2_.

### Cell transfection

The sh-negative control (sh-NC), sh-ZDHHC19, miR-940 mimics, mimics-NC, miR-940 inhibitor and inhibitor NC were obtained from GenePharma. All the steps were conducted as manufacturer’s instruction [18].

### Public dataset analysis

The gene expression profile and clinical data were downloaded from the Therapeutically Applicable Research To Generate Effective Treatments (TARGET) public database.

### RNA extraction and quantitative real-time PCR (qRT-PCR)

Total RNAs were isolated by TRIzol (Invitrogen, CA) and reversely transcribed to complementary DNA with the Reverse Transcription System Kit (Takara, China). The qRT-PCR assay was conducted using the SYBR Green PCR Kit (Takara, China). GAPDH and U6 were used as endogenous controls for mRNAs and miRNAs. All the data were calculated via the 2^−ΔΔCt^ method. The primer sequences used in this study are shown in Supplementary Table S1.

### Cell proliferation, invasion and migration assays

The ability of cell proliferation was evaluated by EDU (Ruibo Bio, China), CCK8 (Dojindo Molecular Technologies, Japan) and colony formation assays. The cell invasion ability was measured using Matrigel invasion chambers (pore size, 8 µm; BD Biosciences). Wound-healing assays were used to determine cell migration capability. All the steps were performed according to the manufacturer’s direction [19].

### Luciferase reporter assay

The luciferase reporter vector containing wt-ZDHHC19 and mut-ZDHHC19 was purchased by GenePharma, China. Cells were separated into three groups: mimics NC & wt-ZDHHC19; mimics miR-940 & wt-ZDHHC19; mimics miR-940 & mut-ZDHHC19. After 48 hours transfection, dual-luciferase system was applied to measure the relative luciferase activity as reported previously [20]. Renilla luciferase was used as an internal reference.

### Animal experiments

Four- to five-week-old BALB/c nude mice were used to structure tumor xenografts of 143B cells. 143B cells transfected with sh-NC or sh-ZDHHC19 were gathered and infected into the mice at a density of 1 × 10^7^ cells per mouse. The relative fluorescence activity, tumor volume and weight were assessed each week. Five weeks after injection, the mice were killed and tumors were collected for further study. Each group contained six mice. The animal experiments were approved by the Experimental Animal Ethics Committee of Zhengzhou University.

### Western blot

Protein was collected using the RIPA lysis reagent (Biyuntian, China) with 1% PMSF (Invitrogen Life Technologies) and the concentration was measured through BCA method (Solarbio, Beijing China). Approximately 50 ug of protein from each sample was loaded on the 12% SDS-PAGE gel and then transferred onto a PVDF membrane (Sigma, USA) after electrophoresis. The membrane was then blocked in the 5% skimmed milk powder and incubated with the corresponding primary antibody at 4°C overnight. After being washed with PBST, the IRDye® goat anti-mouse or anti-rabbit secondary antibodies (Odyssey LI-COR, USA) were provided for 1 h at room temperature. The membrane was imaged by an Odyssey imaging system. The primary antibodies used in this experiment are as follows: ZDHHC19 (1:1500, CST, USA); wnt3a (1:1500, CST, USA); β-catenin (1:1500, CST, USA); APC (1:1000, CST, USA); cyclinD1 (1:1000, CST, USA); MMP2 (1:1000, CST, USA); MMP7 (1:1000, CST, USA); MMP9 (1:1000, CST, USA) and GAPDH (1:1500, CST, USA).

### Immunohistochemistry (IHC) analysis

Immunohistochemistry staining was conducted as reported previously [21]. Briefly, the section was dewaxed, and the antigen was restored in EDTA buffer. Then, the section was incubated with 3% H_2_O_2_ in order to inhibit endogenous peroxidase. After blocking with 5% goat serum, the corresponding primary antibody was added onto the section at 4°C overnight, and then the section was incubated with the second antibody (Dako, Danish). After being treated with the DAB Detection Kit (Dako, Danish) and hematoxylin, two pathologists who were blind to the clinical information were invited to score the samples according to their staining intensity and the percentage of positive cells from 1 to 5. The score from 1 to 3 was regarded as a low expression group and the score from 4 to 5 was regarded as a high expression group. The primary antibodies used in this experiment are as follows: ZDHHC19 (1:200, CST, USA); Ki-67 (1:200, CST, USA).

### Bioinformatics analysis

TARGET online database was used for bioinformatics analysis. Gene Set Variation Analysis (GSVA), Gene Set Enrichment Analysis (GSEA) and Kyoto Encyclopedia of Genes and Genomes (KEGG) analysis was performed to predict the potential mechanism of ZDHHC19 in OS. All the steps were finished through RStudio.

### Statistical analysis

All statistical analysis were conducted using SPSS 23.0 (IBM, Chicago, USA) and GraphPad Prism 8 (Inc., La Jolla, USA). Student’s *t* test was used for the comparison between two groups. A one-way ANOVA analysis was applied to assess the differences in three or more groups. Kruskal–Wallis test was performed to evaluate the grade data. The prognostic value of ZDHHC19 was determined by Kaplan–Meier analysis. All data was presented as mean ± SD, and *P* < 0.05 was considered to be of statistical significance.

## Results

### ZDHHC19 was overexpressed and correlated with poor prognosis in OS

**Figure 1.**
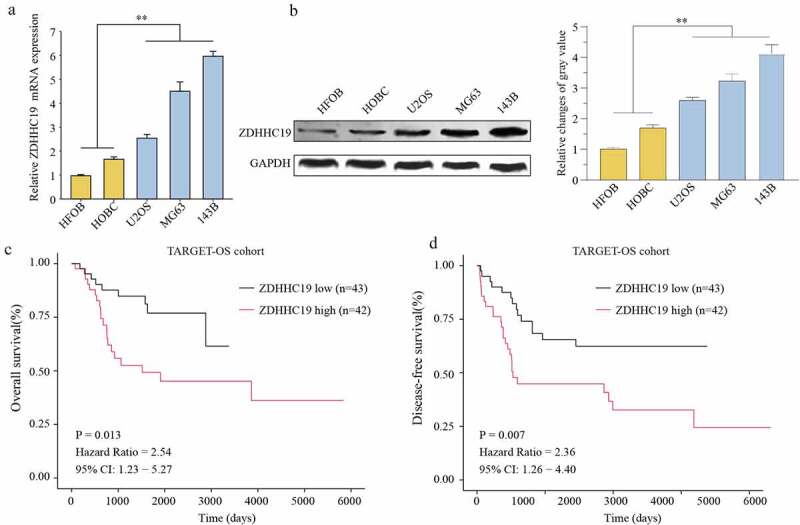
ZDHHC19 was upregulated in OS and predicted poor prognosis. The expression status of ZDHHC19 in OS cell lines (U2OS, MG63 and 143B) and the normal human bone cells (HFOB and HOBC) at mRNA (a) and protein levels (b) were determined by RT-qPCR and Western blot, respectively. Kaplan-Meier survival curves of overall survival (c) and disease-free survival (d) between high ZDHHC19 expression group and low ZDHHC19 expression group in TARGET database. *P < 0.05; **P < 0.01.

We first explored the expression pattern of ZDHHCs in OS using the GSE126209 database and Western blot assay (Supplementary Figure S1), and we found several ZDHHCs were dysregulated expression in OS tissues. To further identify ZDHHC19 expression in OS, we performed qRT-PCR assay to examine the ZDHHC19 expression profiles in cell lines, and found ZDHHC19 expression level was dramatically enhanced in OS cell lines (U2OS, MG63 and 143B) compared with normal bone cells (HFOB and HOBC) by qRT-PCR and Western blot (Figure 1(a,b)). Moreover, the results of TARGET database analysis revealed that overall survival and disease-free survival were both shorter in patients with ZDHHC19 high expression compared with those with ZDHHC19 low expression (Figure 1(c,d)). In summary, ZDHHC19 was overexpressed in OS and positively associated with poor clinical outcomes.

### ZDHHC19 silencing inhibited proliferation, invasion and migration of OS cells in vitro

**Figure 2.**
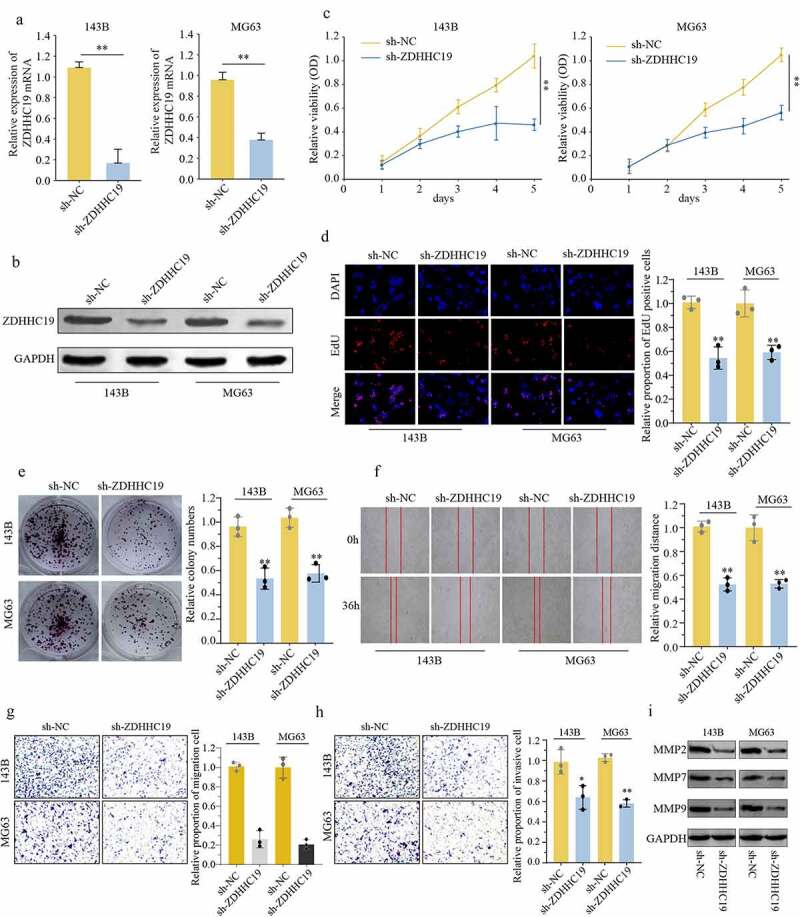
ZDHHC19 knockdown inhibited cell proliferation, invasion and migration in OS cells. (a, b) qRT-PCR and Western blot analysis of OS cells transfected with sh-ZDHHC19 or sh-NC. The ability of proliferation in OS cells transfected with sh-ZDHHC19 or sh-NC by CCK8 (c), EDU (d) and colony formation (e). (f) Analysis the effect of ZDHHC19 on the migration of 143B and MG63 cells by wound-healing assay. The ability of invasion and migration in OS cells transfected with sh-ZDHHC19 or sh-NC by transwell migration (g) and transwell invasion assay (h). (i) Expressions of migration-related proteins (MMP2, MMP7 and MMP9) in sh-ZDHHC19-transfected OS cells were detected by Western blot analysis. All data are presented as the mean ± standard deviation of three independent experiments. *P < 0.05; **P < 0.01.

To further explore the action of ZDHHC19 in OS, 143B and MG63 cells were transfected with sh-ZDHHC19 or sh-Negative Control (sh-NC) lentivirus. qRT-PCR and Western blot were used to verify the transfection efficacy (Figure 2(a,b)). CCK8 and EDU assays were applied to evaluate the role of ZDHHC19 on the proliferation of OS cells. Results showed that the growth ability of cells transfected with sh-ZDHHC19 was obviously decreased as compared to sh-NC (Figure 2(c,d)). Congruously, colony formation assay also showed that ZDHHC19 silencing could suppress cell proliferation (Figure 2(e)). Meanwhile, TUNEL assay indicated that apoptosis rate was increased following ZDHHC19 silencing (Supplementary Figure S2). Interestingly, ZDHHC19 silencing did not affect the proliferation ability of non-tumor cells (Supplementary Figure S3). Moreover, downexpression of ZDHHC19 remarkably impeded the migration and invasion ability of 143B and MG63 cells by wounding healing assay, transwell and invasion assay (Figure 2(f-h)). Furthermore, the expression levels of invasion and migration-related proteins (MMP2, MMP7 and MMP9) were detected. Expressions of MMP2, MMP7 and MMP9 were decreased following ZDHHC19 knockdown (Figure 2(i)). Above all, all data indicated that ZDHHC19 silencing could inhibit OS malignant progression *in vitro*.

### Knockdown of ZDHHC19 impaired OS growth and metastasis in vivo

**Figure 3.**
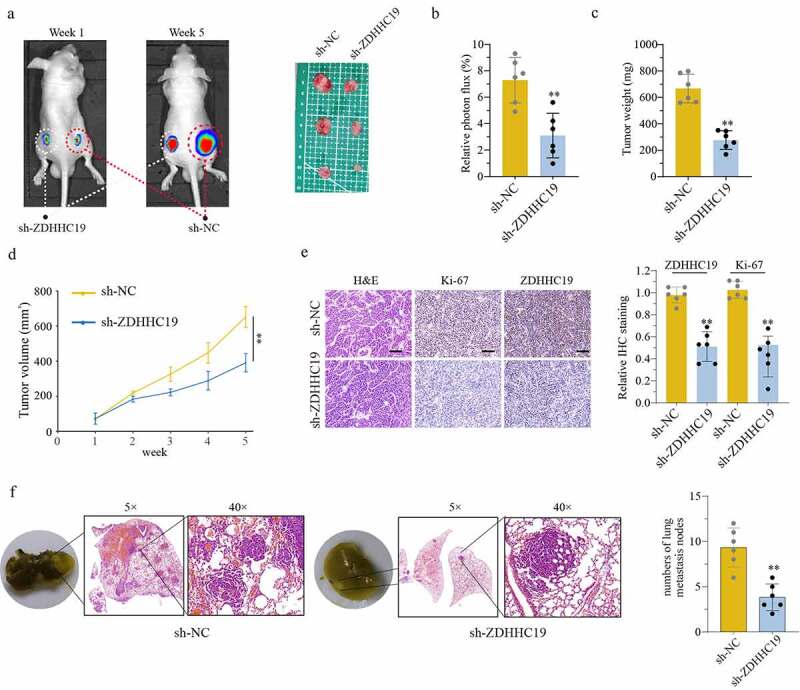
ZDHHC19 knockdown suppress tumor growth *in vivo*. (a) The tumor representative image from mice injected with OS cells transfected with sh-ZDHHC19 or sh-NC. (b) Relative photon flux analysis between sh-ZDHHC19 group and sh-NC group. (c) Tumor weight analysis between sh-ZDHHC19 group and sh-NC group. (d) Tumor volume analysis between sh-ZDHHC19 group and sh-NC group. (e) The representative staining images and expression levels analysis of ZDHHC19 and Ki-67 in tumors from sh-ZDHHC19 group and sh-NC group. (f) The representative images and quantitative analysis of lung metastasis between sh-ZDHHC19 and sh-NC group. All data are presented as the mean ± standard deviation of three independent experiments. *P < 0.05; **P < 0.01.

In order to determine whether ZDHHC19 could promote tumor growth *in vivo*, 143B cells transfected with sh-ZDHHC19 or sh-NC were injected into nude mice, respectively. In accordance with the results of *in vitro* experiments, there was a significant inhibition in sh-ZDHHC19 group compared with the sh-NC group, including the reduction in relative photon flux, tumor weight and tumor volume (Figure 3(a-d)). In addition, the IHC data revealed that ZDHHC19 and Ki-67 protein expression were weaker after ZDHHC19 silencing, indicating that the proliferative ability of tumor was impaired (Figure 3(e)). Meanwhile, the ability of lung metastasis was remarkably inhibited after ZDHHC19 knockdown (Figure 3(f)). Taken together, all these suggested that ZDHHC19 could accelerate tumor growth and metastasis *in vivo*.

### ZDHHC19 activated wnt/β-catenin pathway in OS

**Figure 4.**
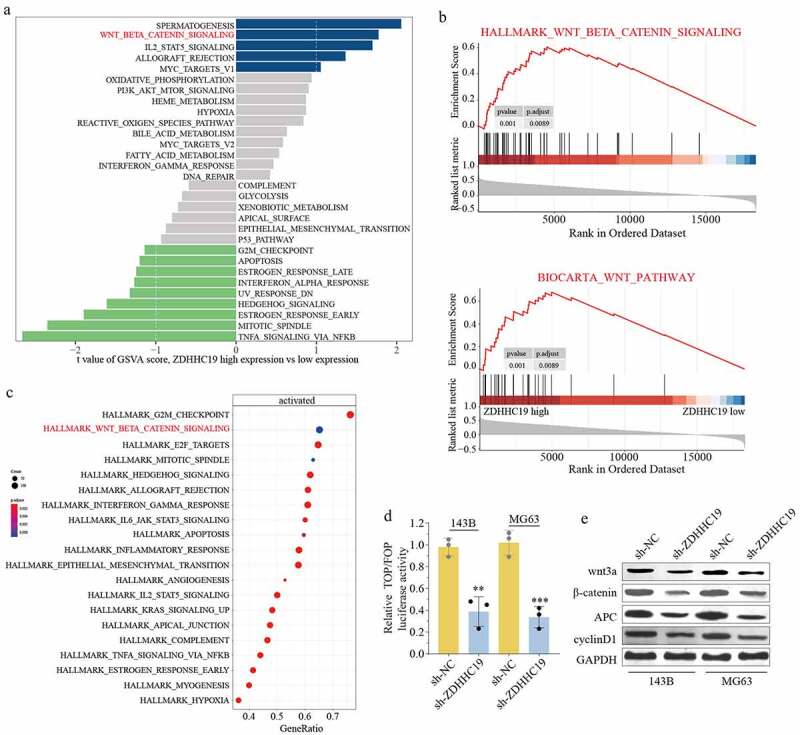
ZDHHC19 promoted OS progression through wnt/β-catenin signaling pathway. (a) GSVA analysis showed the enrich pathways between ZDHHC19 high expression group and low expression group. (b) GSEA analysis suggested the significant correlation between wnt/β-catenin signaling and ZDHHC19 expression. (c) KEGG analysis showed enriched pathways in TARGET-OS cohort. (d) The relative wnt/β-catenin signaling activity analysis between sh-ZDHHC19 group and sh-NC group. (f) Western blot analysis of wnt/β-catenin signaling related proteins between sh-ZDHHC19 group and sh-NC group. All data are presented as the mean ± standard deviation of three independent experiments. *P < 0.05; **P < 0.01.

In accordance with previous sequencing results, the p-STAT3 expression level was remarkably decreased following ZDHHC19 silencing in OS cells (Supplementary Figure S4). To gain insight into the underlying mechanism of ZDHHC19 in OS, we further explored its downstream pathway. Through bioinformatics analysis, we demonstrated a positive correlation between wnt/β-catenin signaling pathway and high ZDHHC19 expression (Figure 4(a-c)). Further, a TOP-Flash luciferase assay was conducted to verify this speculation. As shown in Figure 4(d), the relative luciferase activity was reduced in sh-ZDHHC19 group, suggesting that ZDHHC19 served a crucial role in activating wnt signaling. Meanwhile, the expression of wnt/β-catenin pathway proteins, such as wnt3a, β-catenin, APC and cyclinD1, was also decreased after ZDHHC19 knockdown 120 hours (Figure 4(e)). Collectively, these data strengthened the verdict that ZDHHC19 facilitated the activation of wnt/β-catenin pathway in OS.

### ZDHHC19 was a target of miR-940

**Figure 5.**
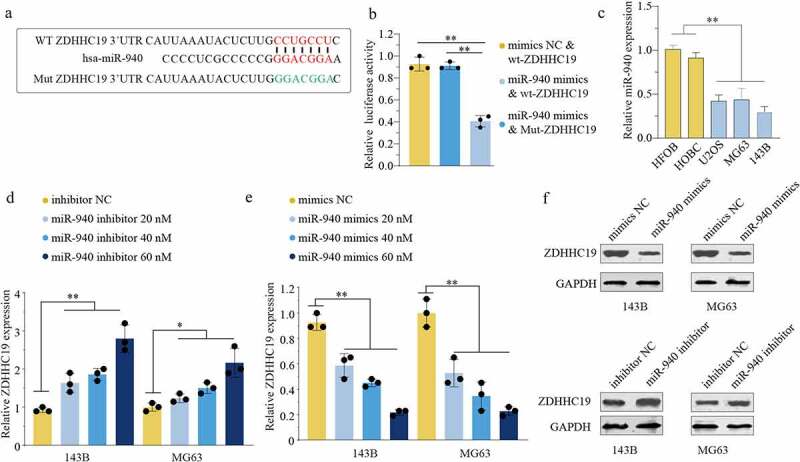
miR-940 directly bound to the 3’-UTR of ZDHHC19 and reversely regulated ZDHHC19 expression. (a) Bioinformatics analysis showed the miR-940 potential binding site located on the 3’-UTR of ZDHHC19. (b) The relative luciferase analysis based on a dual – luciferase reporter assay. (c) The expression status of miR-940 in OS cell lines (U2OS, MG63 and 143B) and the normal human bone cells (HFOB and HOBC). (d) The mRNA expression level of ZDHHC19 in OS cells transfected with inhibitor NC or miR-940 inhibitor. (e) The mRNA expression level of ZDHHC19 in OS cells transfected with mimics NC or miR-940 mimics. (f) The protein expression level of ZDHHC19 in OS cells transfected with miR-940 inhibitor or miR-940 inhibitor. All data are presented as the mean ± standard deviation of three independent experiments. *P < 0.05; **P < 0.01.

Furthermore, we investigated the upstream regulation mechanism of ZDHHC19 and found that ZDHHC19 might be a potential target of miR-940 based on online databases. Figure 5(a) shows the potential-binding site by bioinformatic analysis. The luciferase reporter assay was applied to confirm the direct binding and the results disclosed that miR-940 overexpression suppressed luciferase activity in wt-ZDHHC19 group but not mut-ZDHHC19 group, indicating that miR-940 could directly bind to 3’-UTR of ZDHHC19 (Figure 5(b)). Contrary to the upregulated expression status of ZDHHC19, OS cell lines exhibited low miR-940 expression levels (Figure 5(c)). Meanwhile, ZDHHC19 expression was enhanced after transfection with miR-940 inhibitor, whereas decreased with miR-940 overexpression in OS cells both in mRNA (Figure 5(d,e)) and protein levels (Figure 5(f)). These findings revealed that miR-940 could directly bind to the 3’-UTR of ZDHHC19 and negatively regulate its expression.

### miR-940 suppressed OS progression by downregulating ZDHHC19

**Figure 6.**
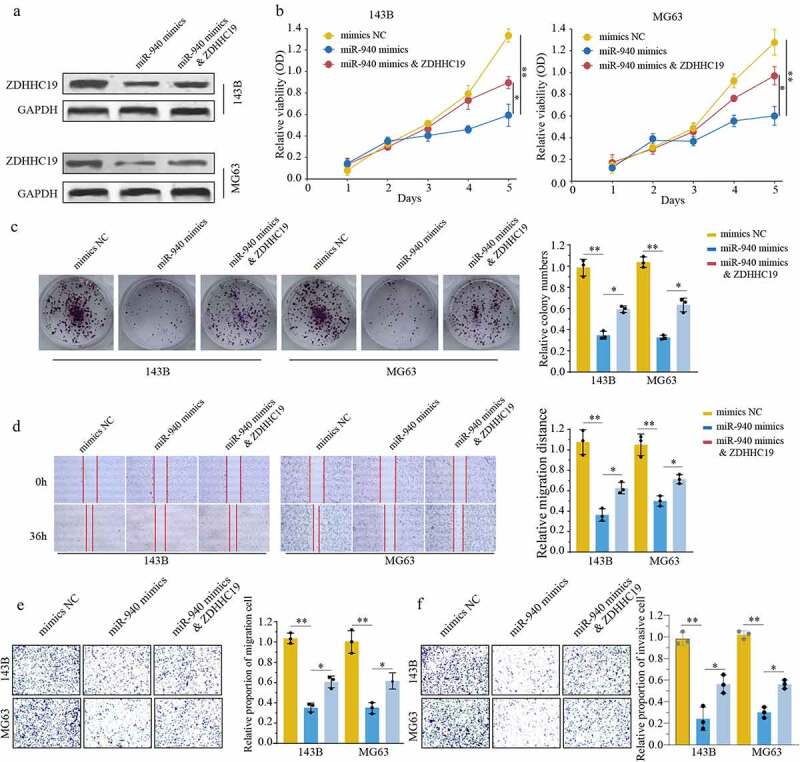
ZDHHC19 overexpression could partly reverse the inhibitory effect of miR-940 in OS progression. (a) The protein expression level of ZDHHC19 in OS cells transfected with mimics NC, miR-940 mimics or miR-940 mimics & ZDHHC19 plasmid. (b, c) The ability of cell proliferation in OS cells transfected with mimics NC, miR-940 mimics or miR-940 mimics & ZDHHC19 plasmid by CCK8 (b) and colony formation (c). (**D, E, F**) The ability of migration in OS cells transfected with mimics NC, miR-940 mimics or miR-940 mimics & ZDHHC19 plasmid determined by wound healing assay (d), transwell migration (e) and transwell invasion assay (f). All data are presented as the mean ± standard deviation of three independent experiments. *P < 0.05; **P < 0.01.

The above consequences reminded us to investigate the biological function of miR-940/ZDHHC19 axis in osteosarcoma. We divided our experiments into three groups as follows: cells transfected with mimics NC, transfected with miR-940 mimics and transfected with miR-940 mimics & ZDHHC19 plasmid. The transfection efficiency was verified by Western blot (Figure 6(a)). As shown in Figure 6(b,c), there was a remarkably impaired proliferation of OS cells after transfection with miR-940 mimics, and ZDHHC19 overexpression could reverse this inhibition partly. The wound healing assay showed that the enforced expression of ZDHHC19 could partially reverse the negative effects on cell wound healing activity induced by miR-940 (Figure 6(d)). Meanwhile, the ability of invasion and migration was also suppressed after transfected with miR-940 mimics and restored partly by ZDHHHC19 upregulation (Figure 6(e,f)). Overall, these results demonstrated that miR-940 suppressed OS progression by downregulating ZDHHC19.

### miR-940/ZDHHC19 axis regulates wnt/β-catenin pathway

**Figure 7.**
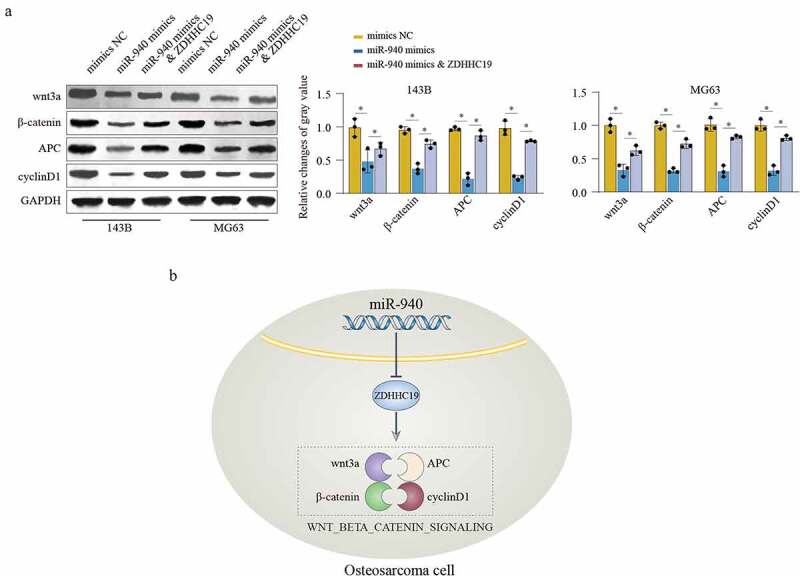
miR-940/ZDHHC19 axis regulates wnt/β-catenin pathway. (a) Western blot analysis of wnt/β-catenin signaling related proteins in OS cells transfected with mimics NC, miR-940 mimics or miR-940 mimics & ZDHHC19 plasmid. All data are presented as the mean ± standard deviation of three independent experiments. *P < 0.05; **P < 0.01. (b) Summary of the mechanism by which miR-940/ZDHHC19 axis regulates the malignant phenotype of OS.

We further evaluated the function of miR-940/ZDHHC19 axis on wnt/β-catenin signaling. The results suggested that miR-940 overexpression notably inhibits the expression level of the wnt/β-catenin signaling-related proteins, containing wnt3a, β-catenin, APC and cyclinD1. Meanwhile, ZDHHC19 reintroduction partially rescued the inhibition effects on wnt/β-catenin signaling in miR-940 overexpression group at 120 hours (Figure 7(a)). Taken together, these findings demonstrate that miR-940 inhibit OS cell proliferation and invasion partially through ZDHHC19 mediated wnt/β-catenin pathway activation (Figure 7(b)).

## Discussion

OS contributes to be the leading cause of cancer-related mortality in primary bone tumor [22]. Although multiple risk factors in OS have been verified, their precise pathogenesis remains unclear. Hence, it is extremely crucial to disclose the molecular mechanism underlying osteosarcoma progression.

In our research, we revealed that ZDHHC19 was upregulated in OS cell lines. Moreover, the high expression of ZDHHC19 was correlated with poor prognosis, indicating that ZDHHC19 might participate in the development of OS. Furthermore, functional experiments and mechanism exploration suggested that ZDHHC19 promoted OS proliferation and metastasis *in vitro* and *in vivo* through activating wnt/β-catenin signaling pathway. Consistent with our results, Niu *et al*. determined that ZDHHC19 was overexpressed in lung squamous cell carcinomas and could promote cancer progression through active STAT3 activity by S-Palmitoylation [8]. Moreover, ZDHHC11 mRNA expression was elevated in Burkitt lymphoma cells, and it could accelerate tumor proliferation [23]. It was reported that ZDHHC17 was upregulated in glioblastoma multiforme (GBM) and contributed to GBM malignant development through promoting MAP2K4 and P38/JNK activation [24]. Patients with high ZDHHC18 expression had poor overall survival rate compared with those with low ZDHHC18 expression in renal cancer, liver cancer and glioma [25]. Overall, ZDHHC19 served as an oncogene in OS progression.

Emerging research studies have demonstrated that miRNAs can mediate mRNA expression by interacting with the binding sites in the 3’-UTR of mRNAs and that the miRNAs-mediated dysregulation of mRNAs can cause epigenetic alteration and tumorigenesis [26]. For example, miR-654-5p could suppress OS cell proliferation and metastasis by targeting SIRT6 [27]. MiR-1225-5p served as a tumor inhibitor in OS by downregulating Sox9 [28]. However, whether any miRNAs could participate in the dysregulation of ZDHHC19 in OS has not been reported up to now. In this study, bioinformatics analysis was used to identify miRNAs involved in the regulation of ZDHHC19 expression in OS cells, and the results indicated that there was a potential binding site between miR-940 and ZDHHC19. The results of qRT-PCR and Western blot disclosed that there was a negative association between miR-940 and ZDHHC19. The luciferase reporter assay verified that miR-940 could directly bind to the 3’-UTR of ZDHHC19. Similarly, it was reported that miR-940 could reversely regulate CKS1 [29], MACC1 [30], MTHFD2 [31] and SPOCK1 [32] in tumors, indicating its significant function in tumor occurrence and development.

Previous researches have demonstrated that the weak expression of miR-940 is regularly verified in multiple tumors such as cervical cancer [33], non-small cell lung cancer [34], hepatocellular carcinoma [35] and so on. In the current study, we found that miR-940 was downregulated in OS tumor tissue specimen compared with normal tissue samples. Furthermore, miR-940 could inhibit OS cells proliferation and invasion through downregulating ZDHHC19 expression. Coincidently, miR-940 also acted as a tumor suppressor and was involved in multiple tumor progression. For example, miR-940 was downregulated in esophageal squamous cell carcinoma and correlated with advanced clinical stage, poor differentiation, lymph node metastasis and poor prognosis. Meanwhile, miR-940 overexpression could decrease cell viability, arrest cell cycle and promote apoptosis [36]. Wang *et al*. also determined that ectogenous miR-940 expression suppress cell growth in ovarian cancer [37]. However, some researchers also verified that miR-940 promoted tumor development in gastric cancer and endometrial cancer, suggesting the complicated function of miR-940 in tumors, which needs further exploration.

## Conclusion

For the first time, we identified that ZDHHC19 was overexpressed in OS and associated with worse prognosis. Functional experiments have verified that ZDHHC19 could promote cell proliferation, invasion and migration *in vitro* and *in vivo* through activating wnt/β-catenin pathway. Mechanism exploration determined that miR-940 could bind to the 3’-UTR of ZDHHC19 and reversely regulated its expression. Overall, miR-940/ZDHHC19 axis contributed to OS progression and might be considered as a novel target for OS treatment.

## Supplementary Material

Supplemental Material
