# Supplementary material for: Zinc finger Asp-His-His-Cys palmitoyl -acyltransferase 19 accelerates tumor progression through wnt/β-catenin pathway and is upregulated by miR-940 in osteosarcoma
Source: Bioengineered. 2022 Mar 17;13(3):7367–79. doi: 10.1080/21655979.2022.2040827 (PMC9278973; doi:10.1080/21655979.2022.2040827)
Supplement: Supplemental Material [file KBIE_A_2040827_SM5944.zip › supplementary/supplementary Table 1.docx]

**Supplementary Table 1. Primer sequence used in this study**

| **siRNA** | **sense sequence** |
| --- | --- |
| miR-940 forward primer | 5'- ACACTCCAGCTGGGAAGGCAGGGCCCCCG -3' |
| miR-940 reverse primer | 5'- CTCAACTGGTGTCGTGGAGTCGGCAAT  TCAGTTGAGGGGGAGCG -3' |
| U6 forward primer | 5'-CCAGUUUACCUAACGCAAUTT-3' |
| U6 reverse primer | 5'-TTCACGAATTTGCGTGTCAT-3' |
| GAPDH forward primer | 5′-ACCAGGAAATGAGCTTGACA-3′ |
| GAPDH reverse primer | 5′-GACCACAGTCCATGCCATC-3′ |
| ZDHHC19 forward primer | 5′- TTGCTGCCTTCAATGTGGTG -3′ |
| ZDHHC19 reverse primer | 5′- CGGAGCCTTGATGTAAGATGC -3′ |
