# Supplementary material for: Zinc finger Asp-His-His-Cys palmitoyl -acyltransferase 19 accelerates tumor progression through wnt/β-catenin pathway and is upregulated by miR-940 in osteosarcoma
Source: Bioengineered. 2022 Mar 17;13(3):7367–79. doi: 10.1080/21655979.2022.2040827 (PMC9278973; doi:10.1080/21655979.2022.2040827)
Supplement: Supplemental Material [file KBIE_A_2040827_SM5944.zip › supplementary/supplementary.pdf]

Supplementary materials

Supplementary figure S1

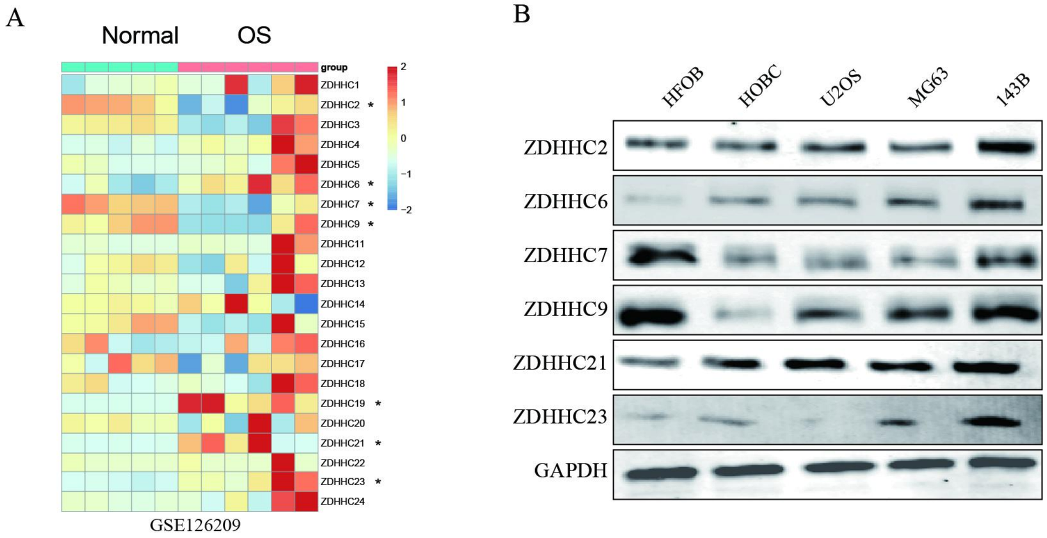

(a) Identification of differentially expressed ZDHHCs family members between osteosarcoma and normal controls in the GEO database (GSE126209). (b) Expression level of ZDHHCs family members in OS cell lines (U2OS, MG63 and 143B) compared with normal bone cells (HFOB and HOBC). *P<0.05;

Supplementary figure S2

(a) Apoptosis rate of 143B and MG63 cells after ZDHHC19 silencing was determined by TUNEL assay. (b) Apoptosis-related proteins (BAX, BAK, cleaved caspase-3) was significantly increased in ZDHHC19 silencing osteosarcoma cells. (c) GSEA analysis suggested the significant correlation between cell cycle and apoptosis signaling and ZDHHC19 expression. *P<0.05;**P<0.01;

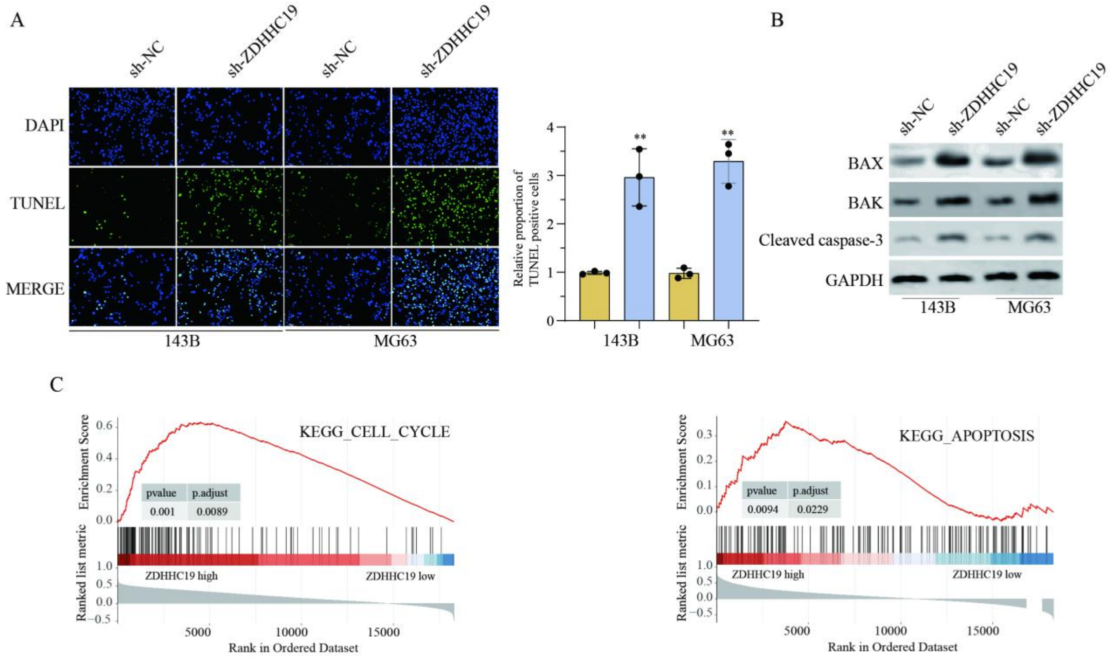

Supplementary figure S3

The ability of proliferation in normal bone cells (HFOB and HOBC) transfected with sh-ZDHHC19 or sh-NC by CCK8 (a) and EDU (b).

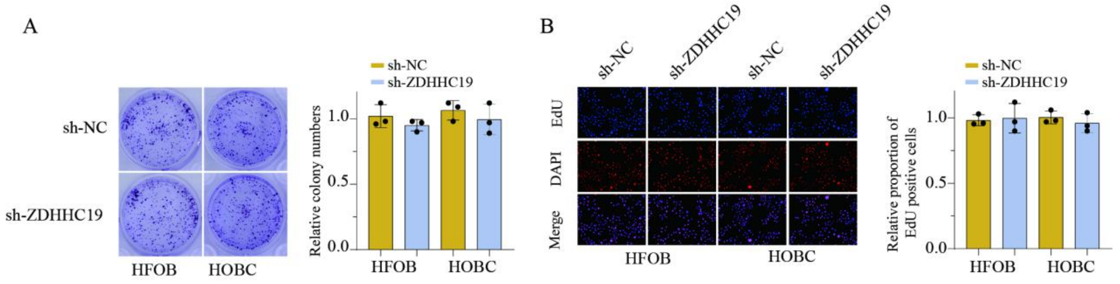

Supplementary figure S4

Western blot analysis of STAT3 and p-STAT3 proteins between sh-ZDHHC19 group and sh-NC group. All data are presented as the mean ± standard deviation of three independent experiments. ***P<0.001.

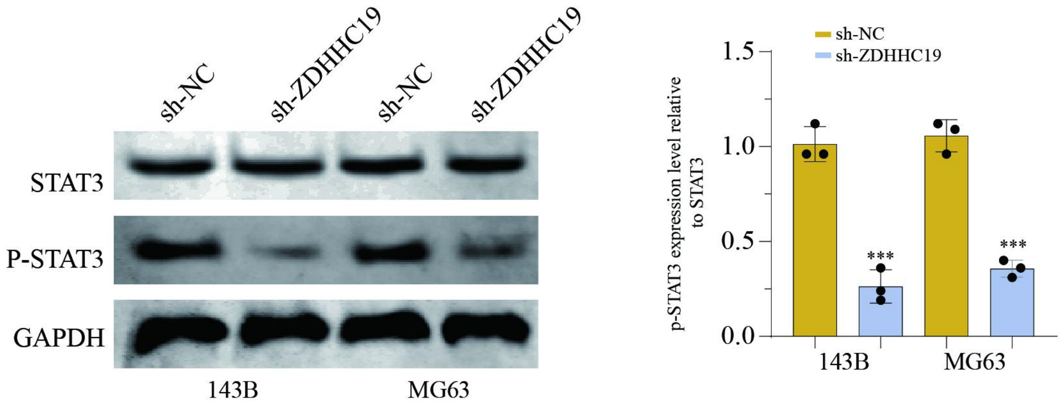
sh-NC
sh-ZDHHC19
sh-NC
sh-ZDHHC19
STAT3
P-STAT3
GAPDH
143B
MG63
sh-NC
sh-ZDHHC19
p-STAT3 expression level relative to STAT3
1.5
1.0
0.5
0.0
***
***
143B
MG63
